# Interventions to improve physical activity in the elderly (a field project in four municipalities)

**Published:** 2012-09-04

**Authors:** Anniek Appelman

**Affiliations:** ProGez, The Netherlands

**Keywords:** frailty, elderly, physical activity, early screening, lifestyle coaching

## Abstract

**Purpose:**

Case finding by using screening on frailty and physical activity among elderly and coaching them on lifestyle and to find appropriate activities in order to increase physical activity and well-being of elderly.

**Theory:**

Several stakeholders in community health services and social services, can detect signs of frailty and poverty of physical activity in their contacts with elderly clients. It is also known that adequate physical activity will positively affect the different area’s of frailty like physical and mental abilities as well as social well-being.

Therefore valid screening on frailty and physical activity and subsequent interventions are important to (positively) affect the negative spiral of becoming more and more frail and dependent on health and social services.

**Method:**

In each municipality a local network of health care professionals and social workers came together in order to make agreements on the process of early screening and lifestyle coaching focused on physical activity and on the stakeholders which should be involved. For each municipality 20–40 elderly can be included in the process of lifestyle coaching with the following inclusion criteria:

- in the age of 55 years or older
- living independent (non-residential)
- frailty score of 4 or more or physical ability-score of 2 or more
- poor physical activity and motivation to improve physical activity.

**Evaluation plan:**

Questionnaire among stakeholders in community health services and social services (for early screening) and among the lifestyle coaches. Structured interviews among elderly who are participating in this field project. Measurements using the GFI-scale for frailty and a scale for physical activity.

**Results and conclusions:**

During the period September 2011 until June 2012 the early screening will be performed using the validated Groninger Frailty index and the lifestyle coaches will select and reach them in order to start a coaching process.

**Experiences until now:**

Starting up early screening by several stakeholders demands a good preparation; involvement in making the local appointments about the process of screening and lifestyle coaching is important. Having a coordinator with an important responsibility for communication is crucial. New connections are realized between social workers and health care professionals. To get other professionals involved for early screening is not easy. Focus on frailty in early screening is altered to physical activity. Success for including elderly differs among the municipalities.

Results from structured evaluation are not available yet. In March 2012 some preliminary results will be presented.

Powerpoint presentation available at http://www.integratedcare.org at congresses – San Marino – programme.

